# Modulatory Role of Reactive Oxygen Species in Root Development in Model Plant of *Arabidopsis thaliana*

**DOI:** 10.3389/fpls.2020.485932

**Published:** 2020-09-16

**Authors:** Xuemei Zhou, Yu Xiang, Chenglong Li, Guanghui Yu

**Affiliations:** Hubei Provincial Key Laboratory for Protection and Application of Special Plants in Wuling Area of China, Engineering Research Centre for the Protection and Utilization of Bioresource in Ethnic Area of Southern China, College of Life Sciences, South-Central University for Nationalities, Wuhan, China

**Keywords:** reactive oxygen species, *Arabidopsis thaliana*, root-stem-cell maintenance and differentiation, root-hair development, cell cycle, aerenchyma formation****

## Abstract

Reactive oxygen species (ROS), a type of oxygen monoelectronic reduction product, have a higher chemical activity than O_2_. Although ROS pose potential risks to all organisms *via* inducing oxidative stress, indispensable role of ROS in individual development cannot be ignored. Among them, the role of ROS in the model plant *Arabidopsis thaliana* is deeply studied. Mounting evidence suggests that ROS are essential for root and root hair development. In the present review, we provide an updated perspective on the latest research progress pertaining to the role of ROS in the precise regulation of root stem cell maintenance and differentiation, redox regulation of the cell cycle, and root hair initiation during root growth. Among the different types of ROS, O_2_^•−^ and H_2_O_2_ have been extensively investigated, and they exhibit different gradient distributions in the roots. The concentration of O_2_^•−^ decreases along a gradient from the meristem to the transition zone and the concentration of H_2_O_2_ decreases along a gradient from the differentiation zone to the elongation zone. These gradients are regulated by peroxidases, which are modulated by the UPBEAT1 (UPB1) transcription factor. In addition, multiple transcriptional factors, such as APP1, ABO8, PHB3, and RITF1, which are involved in the brassinolide signaling pathway, converge as a ROS signal to regulate root stem cell maintenance. Furthermore, superoxide anions (O_2_^•−^) are generated from the oxidation in mitochondria, ROS produced during plasmid metabolism, H_2_O_2_ produced in apoplasts, and catalysis of respiratory burst oxidase homolog (RBOH) in the cell membrane. Furthermore, ROS can act as a signal to regulate redox status, which regulates the expression of the cell-cycle components CYC2;3, CYCB1;1, and retinoblastoma-related protein, thereby controlling the cell-cycle progression. In the root maturation zone, the epidermal cells located in the H cell position emerge to form hair cells, and plant hormones, such as auxin and ethylene regulate root hair formation *via* ROS. Furthermore, ROS accumulation can influence hormone signal transduction and vice versa. Data about the association between nutrient stress and ROS signals in root hair development are scarce. However, the fact that *ROBHC*/*RHD2* or *RHD6* is specifically expressed in root hair cells and induced by nutrients, may explain the relationship. Future studies should focus on the regulatory mechanisms underlying root hair development *via* the interactions of ROS with hormone signals and nutrient components.

## Introduction

In the Earth’s distant past, the rapid accumulation of oxygen in the atmosphere was an important event for the evolution of multicellular molecular processes (Jeltsch, 2013). Oxygen is an essential element of life for all multicellular organisms including plants and animals especially some specific processes in animals (e.g., oxygen circulation blood vessels) and plants (e.g., cell survive in the deepmost position in roots). In the presence of oxygen, the cellular processes characterized by high-speed electron or energy transport inevitably result in the leakage of electrons or energy in the form of molecular oxygen (O_2_), thereby producing reactive oxygen species (ROS) with a higher chemical activity than O_2_. Consequently, ROS are continuously generated during the respiratory processes in aerobic organisms (Apel and Hirt, 2004). In addition, ROS are a primary product of several enzymatic reactions, which have emerged through cellular evolution. The main forms of ROS include singlet oxygen (^1^O_2_), superoxide anion (O_2_^•−^), hydrogen peroxide (H_2_O_2_), and hydroxyl radical (HO^•^) (Mhamdi and Van Breusegem, 2018; Waszczak et al., 2018). Among these, H_2_O_2_ and O_2_^•−^ are the most stable forms of ROS, having a long lifetime—from milliseconds to seconds, whereas the lifetime of singlet oxygen (^1^O_2_) and hydroxyl radical (HO^•^) is shorter, ranging from nanoseconds to microseconds (Waszczak et al., 2018).

ROS are highly reactive and may cause damage to cellular DNA, lipids, and proteins, and they are often implicated in the development of cancer and other diseases (Hossain et al., 2015). However, growing evidence indicates that ROS may play a critical regulatory role in blood-cell development in the larval lymph glands of *Drosophila melanogaster* (Theopold, 2009), resistance to drought stress and pathogen attack (Qi et al., 2018), and lateral root formation in plants (Biswas et al., 2019). Although ROS pose potential risks to certain processes, they also accumulate in plant root cells under normal growing conditions (Dunand et al., 2007). Furthermore, they are pivotal for the normal growth and development of the root. Recent research in the model plant *Arabidopsis thaliana* has provided strong evidence supporting the indispensable role of ROS in plant root development (Yu et al., 2016; Zeng et al., 2017; Kong et al., 2018; Yamada et al., 2018; Tian et al., 2018), and this research will provide reference for sustainable development of agriculture.

## Gradient Distribution of ROS Regulates Root Stem Cell Differentiation

**Figure 1:**
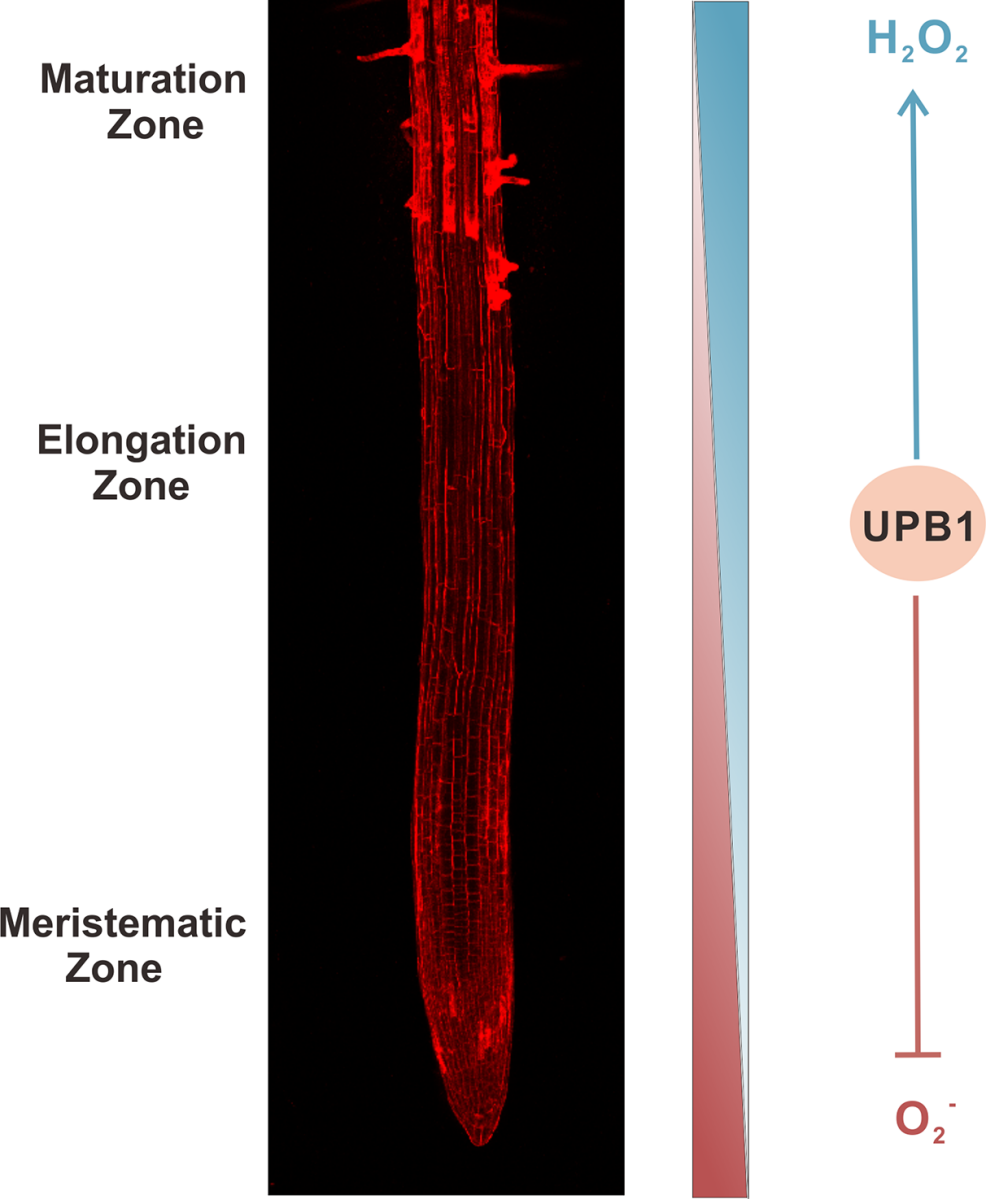
Gradient distribution of superoxide anion (O_2_^•−^) and hydrogen peroxide (H_2_O_2_) in *Arabidopsis thaliana* roots. Nitro blue tetrazolium (NBT) staining showed the distribution of O_2_^•−^ in the roots, from the meristematic to elongation zones. The gradients of reactive oxygen species (ROS) are regulated by peroxidases, which are modulated by the UPBEAT1 (UPB1) factor. The concentration of O_2_^•−^ decreases along a gradient from the meristem to transition zones and the concentration of H_2_O_2_ decreases along a gradient from the differentiation to elongation zones. Arrow-head line indicates stimulation effect, and “T”-shaped line shows the inhibition.

The roots form a key organ that anchors plants to the soil and provides the means to absorb the nutrients and water necessary for plant growth. In addition, roots can sense and respond to changes in the surrounding environment. Root growth relies on the balance of proliferation and differentiation in root stem cells (Petricka et al., 2012). Plant root systems can be divided into three zones along the longitudinal axis; namely, the meristematic, elongation, and maturation zones (Rodriguez-Alonso et al., 2018). The most characteristic stem cells of plants are in the shoot apical meristem and root apical meristem (Sarkar et al., 2007). Stem cells are defined as a specific group of cells with the capacity to self-renew and produce undifferentiated daughter cells, which can form new tissues. Such cells reside in a confined microenvironment known as the stem cell niche, and their characteristics are synergistically maintained by intracellular and extracellular signals (Sarkar et al., 2007). The potential molecular mechanisms underlying the formation and maintenance of plant stem cells have been extensively investigated (e.g., Sarkar et al., 2007; Yang et al., 2018). The role of the synergistic action of transcription factors, regulated by auxins and cytokinins, in the maintenance and differentiation of stem cells has been well established (Singh et al., 2017). Recent research has also revealed that the redox state and the presence of ROS can precisely regulate stem cell fate, and ROS are thus often referred to as a fine-tuner of plant stem cell fate (Tsukagoshi, 2016; Zeng et al., 2017; Yang et al., 2018; Qin et al., 2019). The root tips of *A. thaliana* exhibit complex redox potential patterns, and the quiescent center (QC) and cell regions adjacent to the meristem exhibit the strongest negative potential. The transition and elongation zones are in an oxidized state (Jiang et al., 2016). The implications, function, and regulation mechanism of ROS polarized gradient distribution at root tips are the present highlight research area. Among the different types of ROS, O_2_^•−^ and H_2_O_2_ have been studied more extensively, and they exhibit different gradient distributions in the roots (**Figure 1**) (Dunand et al., 2007; Wells et al., 2010). Their gradient distribution is related to UPBEAT1 (UPB1). UPBEAT1, a basic helix-loop-helix (bHLH) transcription factor, that regulates the expression of a set of peroxidases which participate in the establishment of ROS (H_2_O_2_ and O_2_^•−^) gradient distribution in the root meristem (Tsukagoshi et al., 2010; Perilli et al., 2012; Del Pozo, 2016). This distribution is affected by the nitrate nutrient (Trevisan et al., 2019).

The dynamic balance of ROS in the root apex also plays a key role in modulating cell distribution from the cell division zone to the elongation and maturation (differentiation) zones. O_2_^•−^ and H_2_O_2_ accumulate in the meristematic and elongation zones, respectively (Dunand et al., 2007; Biswas et al., 2019). An imbalance will lead to a change in the size of the meristematic zone. UPB1 regulates the ROS (H_2_O_2_) content in the root apex by inhibiting the expression of class III peroxidases in the elongation zone (**Figure 1**) (Tsukagoshi et al., 2010; Qi et al., 2018). The *upb1-1* mutant in *A. thaliana* presented longer meristems and a lower H_2_O_2_ level in the elongation zone, and the *UPB1* overexpression lines exhibited shorter meristems and a higher H_2_O_2_ level in the elongation zone than those in the wild type. Conversely, the peroxide level in the meristematic zone was higher in the *upb1-1* mutant but lower in the *UPB1* overexpression lines. Furthermore, the overexpression of a *UPB1*-targeted peroxidase resulted longer meristems than those in the wild type, and the overexpression of another peroxidase gene, *PER34*, resulted in a longer-root phenotype than that of the wild type (Tsukagoshi et al., 2010; Tsukagoshi, 2016).

## ROS are Key Regulators of Root Stem Cell Niche Maintenance

**Figure 2:**
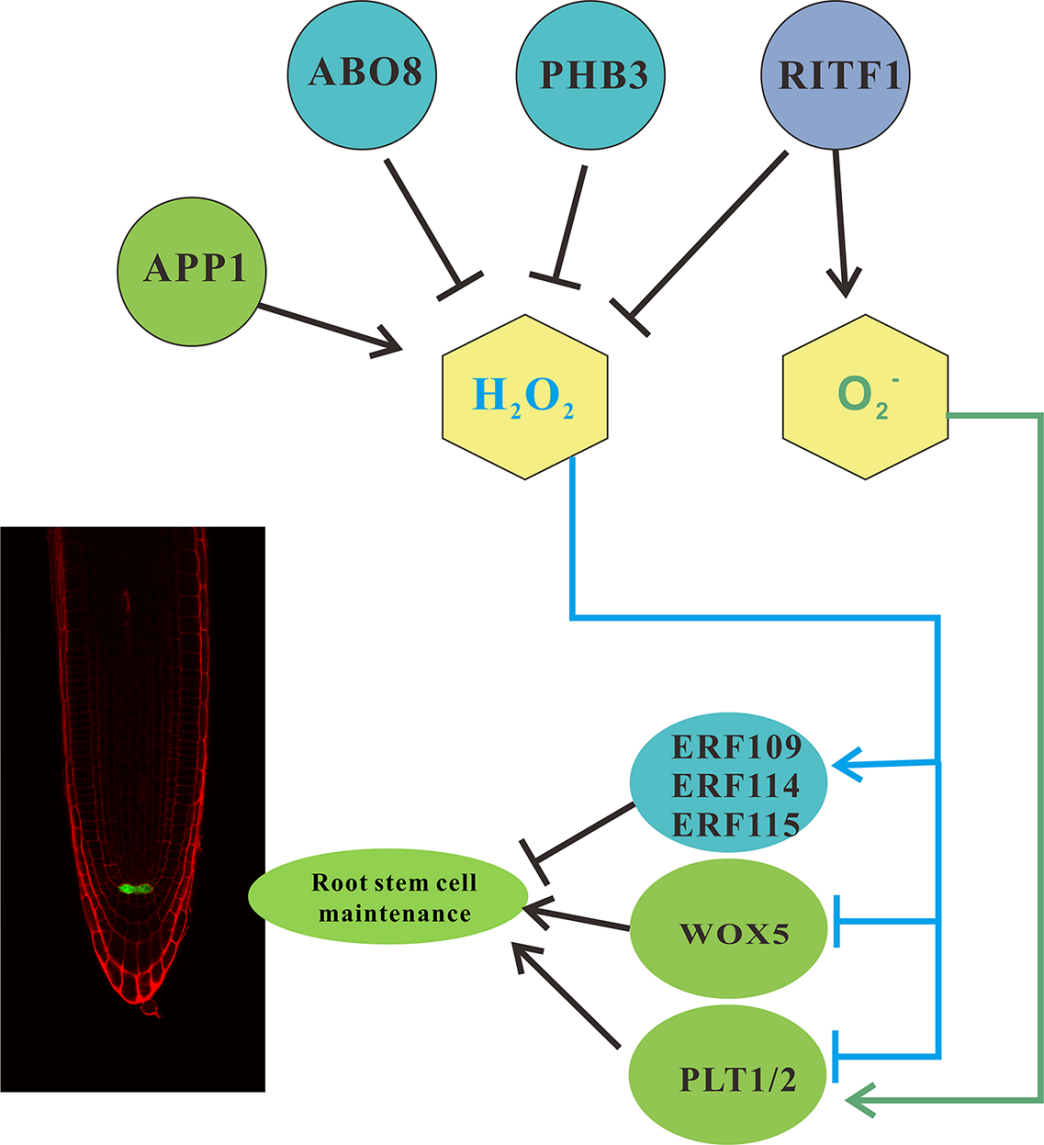
Reactive oxygen species (ROS) mediate root stem cell maintenance. *pWOX5*:*GFP* marks the quiescent center (QC) in *Arabidopsis* roots. Multiple parallel signaling pathways integrate multiple transcription factors to converge as an ROS signal to regulate root stem cell maintenance. APP1 is upregulated, and ABO8 and PHB3 inhibit H_2_O_2_ signals, thus stimulating ERF109/114/115 or inhibiting WOX5. ERF109/114/115 negatively regulates root stem cell maintenance, whereas WOX5 positively regulates it. Another cascade pathway converges on RITF1 to regulate the level of H_2_O_2_ and O_2_^•−^, and its downstream products regulate PLT1/2, thus controlling stem-cell fate. Furthermore, the brassinolide signaling pathway regulates the level of BRAVO and stem-cell fate in the roots. Arrow-head line indicates stimulation effect, and “T”-shaped line shows the inhibition.

There are multiple signal pathways mediated by ROS signals that may be involved in stem cell maintenance and cell fate determination (**Figure 2**). *APP1* encodes a mitochondria-localized P-loop NTPase involving ATP hydrolysis and ROS generation. Loss-of-function alleles of *APP1* caused lower level of ROS (both O_2_^•−^ and H_2_O_2_) in the root meristem, and enhanced the expression of the two peroxidases genes *PER11* and *PER55*, which are involved in ROS detoxification (Del Pozo, 2016). This leads to an increase in the number of cells in the QC and promotes stem cell differentiation. However, *APP1* overexpression leads to defective stem cell niches and higher ROS (H_2_O_2_ and O_2_^•−^) levels in the root meristem (Yu et al., 2016).

Another pathway involves the hormone abscisic acid (ABA). The ABA OVERLY SENSITIVE MUTANT (*ABO8*) gene, encoding a pentatricopeptide repeat domain protein, modulates ROS homeostasis in the root apex (Yang et al., 2014). In the *abo8-1* mutant, ROS accumulates excessively and hinders the expression of *PLETHORA1* (*PLT1*) and *PLT2*, both at the transcriptional and post-transcriptional levels. This leads to the establishment of a hypothetical relationship between ROS signals and *PLT*-mediated maintenance and regulation of the root stem cell niche (**Figure 2**) (Yang et al., 2014; Tsukagoshi, 2016). These results indicate that appropriate ROS levels and gradients play a key regulatory role to preserve the stability of the root stem cell niche (Yu et al., 2016).

Recently, Kong et al. (2018) verified that PROHIBITIN3 (PHB3) maintains the root stem cell niche *via* regulating ROS homeostasis. Transcriptome analysis revealed that some downstream genes including ETHYLENE RESPONSE FACTOR 115 (ERF115), ETHYLENE RESPONSE FACTOR 114 (ERF114), and ETHYLENE RESPONSE FACTOR 109 (ERF109), which are responsible for maintaining the root stem cell niche, were induced by ROS (Yang et al., 2018) (**Figure 2**). In addition, ectopic expression of *ERF115*, *ERF114*, and *ERF109* were found in the *phb3* mutant root meristem, indicating that PHB3 limits the expression of *ERF115*, *ERF114*, and *ERF109* in the root meristem *via* ROS distribution (Kong et al., 2018). Kong et al. (2018) further confirmed that PHYTOSULFOKINE2 (*PSK2*) and *PSK5* are the direct targets of ERF115, ERF114, and ERF109 through ChiP-qPCR assay. Thus, ROS appears to modulate the proliferation of QC cells through the ERF-PSK module (Yang et al., 2018). However, the mechanisms of ROS regulating the expression of PLT1/2, ERF115, ERF114, and ERF109 are still unknown.

The ROOT MERISTEM GROWTH FACTOR 1 (RGF1)-RGFR1/2/3 signaling pathway maintains the characteristics of the root stem cell niche by maintaining the PLT gradients in the proximal meristem (Ou et al., 2016). However, the molecular mechanisms involved in promoting the PLT1/2 protein stability *via* the RGF1-RGFR1/2/3 pathway remain unclear. In a recent study, Yamada et al. (2018) provided evidence that the RGF1-RGFR1/2/3 signaling pathway modulates ROS distribution and enhances PLT1/2 stability. Moreover, PLT2 localization is related to ROS distribution, and transcriptome data analysis of RGF1 treatment revealed that RGF1 INDUCIBLE TRANSCRIPTION FACTOR 1 (RITF1; AT2G12646) is one of the downstream mediators of the RGF1-RGFR1/2/3 pathway (Yamada et al., 2018). This is consistent with the observations in similar ROS distribution phenotypes between *RITF1* overexpression and RGF1-treated roots. The aforementioned results indicate that the RGF1-RGFR1/2/3 signaling pathway maintains the characteristics of the stem cell niche by regulating the ROS levels and distribution by RITF1, and thereby maintaining PLT1/2 stability in the meristematic zone (Yang et al., 2018) (**Figure 2**).

One plausible explanation is that the PLT1/2 stability may be related to ROS-induced post-translational modification. ROS may rapidly modulate the target proteins such as PLT1/2 *via* post-translational modifications, which include phosphorylation, glycosylation, and ubiquitination (Yang et al., 2018). The ROS-sensitive proteins undergo oxidative modifications targeted at sulphur atoms in cysteine and methionine residues in an H_2_O_2_-dependent manner. Research has revealed that H_2_O_2_ treatment of plant cells leads to sulphur oxidation in approximately 100 types of cytosolic proteins (Hossain et al., 2015). Tian et al. (2018) have reported the redox regulation of brassinosteroid (BR) signals, and this process is related to ROS-induced protein modification. BRs induce the generation of H_2_O_2_ in the root meristem, particularly in the root stem cell niche, in a BRASSINOSTEROID INSENSITIVE 1 (BRI1)-dependent manner, and this is required for BRs to promote QC cell division (Yang et al., 2018; Surgun-Acar and Zemheri-Navruz, 2019). *In-vitro* and *in-vivo* studies have confirmed that cys-63 and cys-84 residues are the conserved oxidization sites in BRASSINAZOLE-RESISTANT 1 (BZR1) and BRI1-EMS-SUPPRESSOR 1 (BES1), respectively (Tian et al., 2018). During the oxidative modification of BZR1, the transcriptional activity is enhanced by promoting interactions between BZR1 and key transcriptional regulators of the auxin and light signaling pathways, such as AUXIN RESPONSE FACTOR 6 (ARF6) and PHYTOCHROME INTERACTING FACTOR 4 (PIF4) (Tian et al., 2018).

Mutations in the oxidation sites in the proteins aforementioned such as BZR1 and BES1, or a reduction in endogenous ROS content can significantly impair the functions of BZR1 and BES1 in regulating gene expression and various biological processes, including QC cell division in the roots (Vilarrasa-Blasi et al., 2014; Yang et al., 2018; Surgun-Acar and Zemheri-Navruz, 2019). Furthermore, Vilarrasa-Blasi et al. (2014) indicated that the BRAVO/BES1 signaling model, rather than BZR1, plays a role in BR-mediated stem cell quiescence regulation in plants. In the future, it is worth investigating whether the oxidative modification of BES1, which regulates root stem cell quiescence, leads to changes in BRAVO-BES1 interactions and BRAVO expression.

## Balance of the Intracellular Redox State Fine-Tunes Cell-Cycle Progression

While there is evidence to suggest that ROS regulate the animal cell cycle (Burhans and Heintz, 2009), direct evidence for the role of ROS in the plant cell cycle is still limited. The utilization of exogenous H_2_O_2_ has been reported to inhibit the expression of genes related to cell-cycle inhibition and reduce the size of the root meristem (Tsukagoshi, 2012; Tsukagoshi, 2016). A potential scenario for the accumulation of ROS and prevention of cell proliferation following DNA damage has been reported (Tanaka et al., 2006; Roldán-Arjona and Ariza, 2009). H_2_O_2_ accumulation occurred in the root elongation zone after treatment with zeocin, a double-strand DNA break-inducing agent. The *sog1* mutant was not sensitive to zeocin treatment, and it did not accumulate H_2_O_2_ (Yoshiyama et al., 2009). SUPPRESSOR OF GAMMA RESPONSE 1 (SOG1) is a master transcription factor regulating the response to double-strand DNA break induction (Yoshiyama et al., 2009; Yoshiyama et al., 2013). ChiP-qPCR showed that defense-related genes were the target genes of SOG1, suggesting the involvement of SOG1 in plant immunity (Ogita et al., 2018). *FMO1*, directly controlled by SOG1 under DNA damage conditions, encodes a flavin-containing monooxygenase that is associated with the production of ROS (Chen and Umeda, 2015). Therefore, ROS homeostasis is pivotal in root meristem size modulation following DNA damage. H_2_O_2_ also influences cortex proliferation (Cui et al., 2014).

The redox state regulates the maintenance of the root meristem in plants (Tsukagoshi, 2016). As ROS are highly reactive, the accumulated ROS in cells will oxidize proteins, chemical substances, and metabolites. To prevent such oxidative damage, the cells regulate redox balance through small antioxidant molecules, such as glutathione (GSH) and thioredoxin (TRX) (Hernández et al., 2015; Sevilla et al., 2015). γ-Amino butyric acid (GABA) could function as an antioxidant to scavenge ROS under stress conditions (Liu et al., 2011). In plants, *ROOT MERISTEM LESS 1 (RML1*) encodes the first enzyme in GSH biosynthesis, and active root meristem formation was inhibited in *rml1* mutant plants (Vernoux et al., 2000). The regulatory role of the GSH levels in the G1/S transition of cycling cells has been demonstrated. Glutathione reductase (GR) catalyzes GSH reduction and regulates root meristem maintenance (Schippers et al., 2016). *Arabidopsis thaliana* contains two GR genes, *GR1* and *GR2* (Marty et al., 2009). Based on the T-DNA insertion and homozygous and heterozygous phenotype screening and observation, the complete loss of function of *GR2* leads to embryonic lethality (Tzafrir et al., 2004), severe growth defects were observed in seedlings of *gr2* mutants (Yu et al., 2013).

GSH and TRX also participate in the regulation of root meristem size. Mutants of TRX reductase (*ntra* and *ntrb*) exhibit small meristem phenotypes (Reichheld et al., 2007; Bashandy et al., 2010). These findings provide strong evidence of the key role of cellular redox regulation in maintaining the meristem activity. Redox regulation is a crucial mechanism involving ROS, GSH, GR, and TRX, and it plays an important role in the regulation of plant growth and development. With such a mechanism, hormonal control, energy metabolism, and bioenergetics can be linked to plant growth and development (Schippers et al., 2016). It is highly likely that cell proliferation and differentiation regulated by ROS are affected by the regulation of cell-cycle progression and/or proteins and enzymes involved in cell differentiation, by the coupling of TRX with GSH/GR.

Cell-cycle phases are highly conserved throughout eukaryotic cells; they comprise the G1 phase, which involves DNA unzipping and the start of RNA and protein synthesis, followed by the S phase (DNA synthesis), and G2 phase (lipid synthesis) (Schippers et al., 2016). In these consecutive, dynamic, cellular events, oxygen consumption, energy metabolism, and cellular redox state are closely related with the cell-cycle progression in eukaryotic cells (Burhans and Heintz, 2009; Schippers et al., 2016). Bursts of O_2_^•−^ and H_2_O_2_ activate cell signaling pathways, thereby activating the G0/G1 transition (Kovtun et al., 2000; Diaz Vivancos et al., 2010).

During DNA replication and mitosis in yeast, oxygen consumption and relevant metabolic processes are reduced to their lowest levels. However, it is unclear whether the redox regulation of shoot apical meristematic cell proliferation in plants is similar to relevant mechanisms observed in other eukaryotes. A mechanism conserved in plants and animals is the nuclear localization of GSH during the cell cycle (Diaz Vivancos et al., 2010; García-Giménez et al., 2013). This may be due to reduced auxin polar transport (Bashandy et al., 2010). For instance, reduced polar transport and a weaker auxin response were observed in *grxs17* mutants (Benitez-Alfonso et al., 2009).

The cellular entry of apoplastic H_2_O_2_ is mediated by intrinsic membrane proteins. Although there is no direct evidence of the influence of protein oxidation on cell-cycle components (e.g., cyclins and cyclin-dependent kinases), redox regulation occurs in cell-cycle transcriptional regulators (Schippers et al., 2016). For instance, transcriptional factors, NF-YC (Nuclear Factor-Y subunit C) and TCPs (TEOSINTE BRANCHED/CYCLOIDEA/PCFs) are deactivated *via* cysteine oxidation, and the presence of GSH and GR can reduce such proteins and restore their activity (Schippers et al., 2016). TCPs stimulate the expression of *CYCA2;3*, *CYCB1;1*, and retinoblastoma-related protein (RBR), thereby directly regulating the cell cycle (Schippers et al., 2016). The initial GSH pool may also be induced by the plant hormone jasmonate, and TCPs are negative regulators of jasmonate biosynthesis (Schippers et al., 2016). Therefore, their function will lead to the consumption of the GSH pool, and ultimately causes TCP deactivation through oxidation. In addition, prohibitin is necessary for the coordination of mitochondrial function in the meristem. Lastly, the ROS generated in non-green plastids negatively influence intracellular communication by promoting callose accumulation at plasmodesmata. In the non-green plastids of meristems and organ primordia, the main function of TRX-m3 is to prevent excessive ROS formation (Schippers et al., 2016).

## ROS Regulation During Root Hair Differentiation

Root hairs, which are tubular structures formed by root epidermal cells, facilitate the uptake of nutrients, interaction with microbes, and anchoring of roots to soil (Molendijk et al., 2001). Root hair development comprises four stages: cell specialization, root hair initiation, tip growth, and root hair maturation (Grierson et al., 2014). Epidermal cells are regulated by multiple genes during the specialization process. SCRAMBLED (*SCM*), a leucine-rich repeat receptor-like kinase, allows epidermal cells to sense their location and select the correct cell fate and gene expression patterns. Mutations in this gene disturb the distribution of root hair and non-hair cells (Kwak et al., 2005).

**Figure 3:**
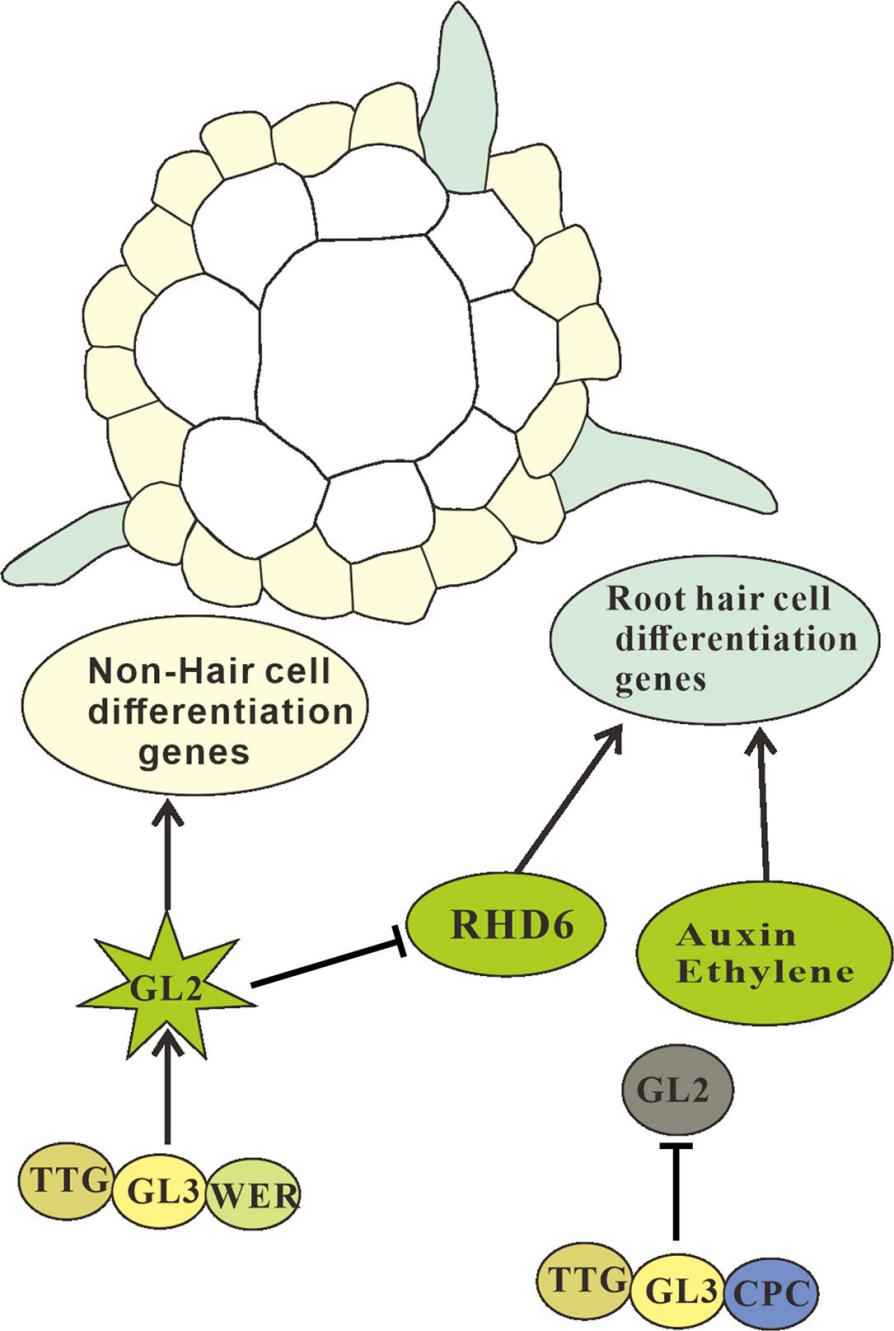
Regulation of crucial components in root-hair development. Transverse section of *Arabidopsis* root showing the relative position of the epidermis and cortex. The epidermis cells are in the H cell position and emerge to form hair cells, and the epidermal cells beneath the cortex cells are in the non-hair cell (N) position and do not form root-hair cells. The root (H) and non-hair cell (N) positions have different components that participate in root-hair formation. Some components shuttle to different types of cells to converge at ROOT HAIR DEFECTIVE (RHD6), a bHLH factor, which when activated, controls the differentiation and formation of root-hair cells, and when non-activated, regulates non-hair-cell formation. Meanwhile, plant hormones such as auxins and ethylene play a positive role in root-hair formation *via* reactive oxygen species (ROS). Arrow-head line indicates stimulation effect, and “T”-shaped line shows the inhibition.

In *A. thaliana*, WEREWOLF *(WER*), TRANSPARENT TESTA GLABRA (*TTG*), and GLABRA3 (*GL3*) simultaneously promote non-hair cell differentiation and inhibit root hair cell differentiation (Galway et al., 1994; DiCristina et al., 1996; Bernhardt et al., 2005). The products of these genes form the WER-GL3/EGL3-TTG complex through physical interactions to positively regulate the expression of GLABRA2 (*GL2*) (AT1G79840) (Bernhardt et al., 2003). *GL2* encodes a homeodomain transcription factor that determines non-hair-cell differentiation by promoting the expression of genes related to non-hair-cell differentiation (DiCristina et al., 1996; Schiefelbein and Lee, 2006) (**Figure 3**).

CAPRICE (*CPC*) encodes a nuclear-localized R3-type MYB transcription factor, which can positively regulate root hair-cell differentiation (Tominaga-Wada et al., 2017). Mutations in this gene result in fewer root hair cells (Wada et al., 1997). CPC can bind with GL3/EGL3-TTG to form an inactive complex, which inhibits *GL2* expression and ultimately promotes epidermal cell differentiation into root hair cells (Tominaga et al., 2007; Song et al., 2011; Kang et al., 2013). Besides *CPC*, other genes that encode R3-MYB proteins include TRIPTYCHON (*TRY*) and ENHANCER OF TRY AND CPC1 (*ETC*) with functions that are partially redundant with those of *CPC* (Schellmann et al., 2002; Kirik et al., 2004; Simon et al., 2007; Serna, 2008; Wang et al., 2016; Tominaga-Wada et al., 2017).

ROOT HAIR DEFECTIVE 6 is a crucial gene encoding a bHLH transcription factor (Menand et al., 2007). Mutations in this gene result in root without root hairs, a condition which can be alleviated with the addition of 1-amino-1-cyclopropanecarboxylic acid or indole-3-acetic acid (IAA) in the medium (Masucci and Schiefelbein, 1994). RHD6-like 4 (RSL4) and MEDIATOR 25 (MED25) also promote root hair elongation and function in the auxin-regulated transcriptional pathway (Foreman et al., 2003; Sundaravelpandian et al., 2013; Mangano et al., 2017).

Polarized growth of root hairs is an ideal model to study the regulation of ROS. NADPH oxidase (NOX), which catalyzes ROS production, and is an effective protein regulating root hair development. NOX, also known as Respiratory Burst Oxidase Homologs (RBOH), plays an important role in plant development (Choudhary et al., 2020; Hu et al., 2020). *RBOHC* (AT5G51060), a member of the Arabidopsis RBOH family, was specifically expressed in Arabidopsis root hairs (Chapman et al., 2019). The study of root hair cells shows that the polarized growth of cells depends on the local accumulation of ROS produced by NADPH oxidase (NOX) (Foreman et al., 2003). Root hairs of ROS mutants without *AtRBOHC*/*RHD2* did not elongate (Foreman et al., 2003).

During root hair formation, owing to changes in the acid environment of the cell wall, cell protrusion is localized to a small disc-shaped area in the cell wall facing outward, approximately 22 µm across, in a process known as root hair initiation (Grierson et al., 2014). Accumulation of large amounts of ROP (Rho of Plant) proteins, which are GTP-binding proteins unique to plants and related to the small GTPases that control the morphogenesis of animal and yeast cells (Vernoud et al., 2003), occur at root hair growth sites (Molendijk et al., 2001). The localization of the ROP proteins is the first marker of root hair formation, and these proteins remain at the tip of developing root hairs throughout root hair growth (Molendijk et al., 2001; Grierson et al., 2014). RHO-RELATED PROTEIN FROM PLANTS 2 (*ROP2*) activates ROS generation through the NADPH oxidase gene ROOT HAIR DEFECTIVE 2 (*RHD2*), which encodes a respiratory burst oxidase homolog (RBOH) or NADPH oxidase (Jones et al., 2007; Gu and Nielsen, 2013). Mutations of this gene impair the ability of ROS to accumulate in the tips of root hairs, thereby inhibiting the development of root hair initials (Foreman et al., 2003). In addition, treating wild-type *A. thaliana* with the NADPH oxidase inhibitor diphenyleneiodonium (DPI) also impairs ROS accumulation in the root tips and leads to the failure of root hair development.

In addition to RHD2 (also called RBOHC), there are nine other respiratory burst oxidase homologs (RBOH), named as RBOHA-RBOHJ (**Table 1**). The isoforms of RBOH regulate all aspects of plant development. For example, *RBOHB*, *RBOHC*/*RHD2*, and *RBOHG* are specific to, or at least relatively highly expressed, in the roots. RBOHC participates in root hair formation and primary root growth, and the mutants of *RBOHC*/*RHD2* exhibit defective root hair phenotypes (Mhamdi and Van Breusegem, 2018). The other RBOH homologs control primary root elongation and lateral root emergence (e.g., RBOHD, RBOHE, and RBOHF) or pollen tube growth (e.g., RBOHH and RBOHJ). The mutants of *RBOHE* and *RBOHH* exhibit reduced fertility and disrupted pollen tube growth (**Table 1**).

**Table 1:** Summary of the role of the respiratory burst oxidase homolog (RBOH) isoforms in plant development.

Gene	Locus tag	Relative expression level	Function(s)	Mutant phenotype
RBOHA	AT5G07390	Specific, highly expressed in the roots and 6–7-week-old siliques	Unknown	Unknown
RBOHB	AT1G09090	Specific, highly expressed in the roots	Seed after ripening	Faster germination of fresh seeds
RBOHC/RHD2	AT5G51060	Specific, highly expressed in the roots	Root hair formation;primary root elongation and development	Root hair defective
RBOHD	AT5G47910	Specific, highly expressed in the cotyledons, hypocotyl, rosette leaves (2–12), cauline, and senescent leaves	Stomata closing, lateral root emergence, and primary root elongation and development	Atypical tubulin formation; early emergence of lateral roots (LRs), and enhanced density of LRs
RBOHE	AT1G19230	Specific, highly expressed in 6–10-week-old of siliques	Anther and pollen development and lateral root emergence	Aborted pollen and reduced fertility
RBOHF/SGN4	AT1G64060	Specific, highly expressed in the stamens and sepals	Stomata closing, lateral root emergence, and primary root elongation and development	Early emergence of lateral roots (LRs) and enhanced density of LRs
RBOHG	AT4G25090	Relatively highly expressed in the roots	Unknown	Unknown
RBOHH	AT5G60010	Specific, highly expressed in mature pollens	Pollen tube growth	Defective root hairs, reduced fertility, and impaired pollen tube growth
RBOHI	AT4G11230	Highly expressed in the roots, and relatively highly expressed in the shoot apex and mature pollens	Unknown	Unknown
RBOHJ	AT3G45810	Specific, highly expressed in mature pollens	Pollen tube growth	Dfective root hairs, reduced fertility, and impaired pollen tube growth

Data were comprehensively analyzed using AtGenExpress eFP, and the excerpt from Mhamdi and Van Breusegem (2018) was obtained with permission granted by the Copyright Clearance Center.

Root hair tip growth is closely related to ROS signaling. ROS accumulation activates calcium channels in root hair cells, increasing the calcium ion levels (Wymer et al., 1997). The Ca^2+^ gradient at the tip of root hairs is a part of the mechanism that regulates growth direction in root hairs, promotes the fusion of vesicles with plasma membranes of root hair tips, and provides raw material for cell wall expansion (Ridge, 1995; Pei et al., 2012). The calcium gradient is maintained in the root hair tips throughout tip growth (Wymer et al., 1997). These results indicate that ROS accumulation in the root hair tips is necessary for normal root hair development (Tsukagoshi, 2016).

## Generation of ROS and Modification of Cell Walls in Root Elongation

The ROS are essential for root growth and development, and one of their major functions in the development of the root system is cell wall modification (O’Brien et al., 2012; Kärkönen and Kuchitsu, 2015). In the root system, the ROS are generated by NADPH oxidases (RBOH) in the plasma membrane or through mitochondrial and plastid respiration (Suzuki et al., 2011; Lázaro et al., 2013; Serrato et al., 2013). The RBOH isoforms may also be key producers of ROS in the apoplast (**Table 1**).

O_2_^•−^ is formed in O_2_ reduction by the catalytic activity of NADPH oxidases (Tsukagoshi, 2016). As the catalytic domain of NADPH oxidases is positioned toward the apoplast, O_2_^•−^ is released into the apoplastic space (Suzuki et al., 2011). Subsequently, O_2_^•−^ is degraded into H_2_O and O_2_ by the catalytic activities of enzymes such as superoxide dismutase (Bowler et al., 1992), apoplastic oxalate oxidases, diamine oxidase, and peroxidase (Federico and Angelini, 1986; Caliskan and Cuming, 1998; Cosio and Dunand, 2009). The H_2_O_2_ generated in the apoplast is then degraded by peroxidases secreted into the apoplastic space (Trevisan et al., 2019).

The shape of plant cells changes with the modification of their cell walls (Grierson et al., 2014). Peroxidases promote the conversion of H_2_O_2_ into H_2_O and O_2_. During this conversion, an electron is also produced and is used to modify the primary and secondary cell walls (Francoz et al., 2015; Tsukagoshi, 2016). The modification process involves electron transfer to lignin monomers, which are subunits of polymeric lignin, in cells in the maturation zone. Upon activation by electrons, lignin monomers will trigger the lignin polymerization process and bind to secondary cell walls during the process of secondary cell wall formation (Novo-Uzal et al., 2013). Lignin in the secondary cell walls provides substantial mechanical strength, which is essential for vascular plants (Ros Barceló, 2005). In addition, NADPH oxidases, peroxidases (e.g., Peroxidase 64 (PER64)), and other enzymes catalyzing ROS metabolism are recruited to form lignin polymerization machinery in the formation of casparian strips (Kamiya et al., 2015), which are bands of lignin that act as diffusion barriers in the endodermal cells of plant roots (Lee et al., 2013; Tsukagoshi, 2016). To facilitate the formation of casparian strips, the casparian strip domain proteins, which are specifically expressed in the endodermis, guide the localization of the aforementioned enzymes into the plasma membrane of endodermal cell walls (Lee et al., 2013; Geldner, 2013).

## ROS Interact With other Signaling Hormones to Regulate Root Development

The ROS act as key signaling molecules under conditions of stress and increasing attention has been paid to the role of ROS in plant stress resistance (Jia, 2011; Gill et al., 2015; Wang et al., 2016). Different environmental stresses, including drought, salt, ultraviolet radiation, and light, can cause an increase in cellular ROS levels (Perez and Brown, 2014; Gururani et al., 2015). ROS accumulation can influence hormone signal transduction, and vice versa (Xia et al., 2015). Auxin, one of the most important plant hormones, influences systematic root development (Bustillo-Avendaño et al., 2018), and participates in meristem maintenance and lateral root formation (Vilches-Barro and Maizel, 2015). Notably, all RBOH transcripts are auxin inducible (Mhamdi and Van Breusegem, 2018).

PLETHORA (PLT) is a key regulator of auxin-induced stem cell niche activity (Aida et al., 2004), and *PLT* expression was altered in *miao* mutants (one kind of Glutathione reductase (GR) mutant) (Yu et al., 2013). Although *PLT2* overexpression in the *miao* mutants does not lead to the recovery of small meristem phenotypes, it increases meristem size in the wild type. Despite the understanding that auxin induces ROS production to regulate cell elongation (Schopfer, 2001) and root gravitropism (Joo et al., 2001), the molecular relationship between ROS and auxin remains largely unknown. Recent study revealed the potential feed-forward loop between ROS and auxin signaling to control lateral root formation (Biswas et al., 2019). It was confirmed that production of reactive oxygen species (ROS) *via* the hormone-induced activation of respiratory burst oxidase homologous NADPH oxidases facilitates lateral root (LR) formation, and that the auxin-induced production of ROS and their downstream products RCS (reactive carbonyl species) modulate the auxin signaling pathway in a feed-forward manner. RCS are key agents that connect the ROS signaling and the auxin signaling pathways (Biswas et al., 2019).

The hormone ABA is a major contributor to the response of plants to abiotic stresses (Nakashima et al., 2014). The accumulation of ABA under abiotic stress conditions reduces root growth. As mentioned above, the production of ABO8 is responsible for splicing NADH dehydrogenase subunit 4 (NAD4) in the mitochondrial complex, and *abo8* mutants were associated with ROS accumulation and ABA production. ROS accumulation was enhanced in the root tips of *abo8* mutants treated with ABA, and this inhibited root growth (Yang et al., 2014). Moreover, auxin distribution and PLT protein levels in the root tip cells of *abo8* mutants were altered. Therefore, ABA-induced ROS accumulation in the mitochondria reduces the root-system growth *via* changes in auxin distribution and PLT levels.

These findings clearly illustrate the complex interactions between plant hormones and ROS in the modulation of root system growth. Other plant hormones such as brassinolide (BR), gibberellin, ethylene, strigolactones, salicylic acid, and jasmonate also participate in hormonal crosstalk (Xia et al., 2015), which in association with ROS, regulate plant growth (Biswas et al., 2019).

Pharmacological and genetic experiments have indicated that auxin and ethylene promote root hair cell differentiation in *A. thaliana*. Treating the roots of *A. thaliana* seedlings with 1-amino-1-cyclopropanecarboxylic acid induced ectopic root hair formation (Tanimoto et al., 1995). In addition, in the ethylene signaling pathway, the CTR1 Raf-like kinase encoded by CONSTITUTIVE TRIPLE RESPONSE (*CTR1*) acts as a negative regulator of root hair formation (Kieber et al., 1993), with mutations in *CTR1* leading to ectopic root hair formation (Dolan et al., 1994; Ikeda et al., 2009). This is consistent with evidence indicating that epidermal cells in the root hair position (H) are more sensitive to ethylene induction than epidermal cells in the non-hair position (N) (Casson and Lindsey, 2003). Besides ethylene and auxin, other hormones also influence root hair development (Konno et al., 2003; Boisson-Dernier et al., 2013). During the early stages of root hair initiation, BRs can influence the fate of root hair cells (Kuppusamy et al., 2009); strigolactones can increase root hair length by interfering with the regulation of cell expansion by auxin, indicating that strigolactones play a role in the late stages of root hair formation (Kapulnik et al., 2011). Similarly, methyl jasmonate promotes root hair growth in a dose-dependent manner, involving the participation of the ethylene and auxin pathways (Zhu et al., 2006).

## Nutrient Stress Regulates Root Hair Development

The major function of root hairs is to expand root surface area, and thus, facilitate water and nutrient uptake from the soil (Grierson et al., 2014). More or longer root hairs are advantageous to plants under low-nutrient conditions. For instance, a high density of long root hairs was more efficient in acquiring phosphate in *A. thaliana* Co and C24 accessions (Narang et al., 2000). Furthermore, under low phosphorus conditions, phosphorus was more efficiently taken up by wild-type plants than the mutants of *rhd6* and *rhd2* (Bates and Lynch, 2000). Enzymes and nutrient transport proteins in root hairs participate in nutrient uptake (Böhme et al., 2004). For example, the activity of ferric chelate reductase (FCR) in wild-type plants was two-fold higher than that in hairless mutants (*rm57/rhd7*), suggesting that this enzyme is localized in the root hairs (Moog et al., 1995).

Root hair development is influenced by nutrient concentrations, and root hair density and length are generally increased under nutrient-deficient conditions (Grierson et al., 2014). Phosphate (Bates and Lynch, 1996), iron (Schmidt et al., 2000), manganese (Konno et al., 2003), and nitrate can increase root hair density in *A. thaliana* (Canales et al., 2017). The density of root hairs in *A. thaliana* ‘Columbia’ grown under low-phosphorus (1.0 µM) conditions was five times greater than that in plants grown under high-phosphorus (1000 µM) conditions (Bates and Lynch, 1996; Savage et al., 2013; Grierson et al., 2014). Under low-phosphorus conditions, the number of root hair-forming files was increased from 8 to 12, and more of the cells in these files formed root hairs than in plants grown under high-phosphorus conditions (Ma et al., 2001; Grierson et al., 2014). Furthermore, the root hairs in *A. thaliana* grown under low-phosphorus conditions were three times longer than those in plants grown under high-phosphorus conditions (Bates and Lynch, 1996). The bHLH transcription factor ROOT HAIR DEFECTIVE6-LIKE4 (RSL4) promotes root hair growth. Thus, the length of root hairs increases in plants grown under low-phosphorus conditions (Yi et al., 2010; Grierson et al., 2014). The same phenomenon was also observed under iron deficiency, which was accompanied with an increase in root hair density and length. In iron-deficient roots, ectopic hairs were produced, and root hair length was doubled (Schmidt et al., 2000). The mechanisms by which different nutrients modulate root hair development differ. For instance, auxin and ethylene signaling is crucial for the responses of plants to iron deficiency, but it has no effect on low-phosphorus responses (Schmidt and Schikora, 2001). Currently, data about the relationship between nutrient stress and ROS signals in root hair development are limited. However, the genes specifically expressed in root hair cells, such as *ROBHC*/*RHD2* and *RHD6*, which may be induced by nutrients, seem to validate this relationship (**Table 1**). A recent study further confirmed that nitrite could affect the expression of UPBEAT1 and localization of ROS in *Zea mays* L. roots (Trevisan et al., 2019).

## ROS Functions in Aerenchyma Formation

The parenchyma tissue with a large number of intercellular spaces is called aerenchyma. Aerenchyma is the evolutionary result of plant adaptation to flood-submerged and waterlogged growth environments (Bailey-Serres et al., 2012; Nishiuchi et al., 2012; Kato et al., 2020), and the classical view is that it is the channel for oxygen to enter the root. For hydrophytes and hygrophytes, aerenchyma forms in their rhizomes; however, terrestrial plants could also differentiate to produce or accelerate the development of aerenchyma in an anoxic environment. In this situation, ROS and ethylene signaling are involved in this adaptation regulation (Yamauchi et al., 2014; Sasidharan and Voesenek, 2015; Singh et al., 2016; Choudhary et al., 2020; Hong et al., 2020). Lysigenous aerenchyma contributes to the ability of plants to tolerate low-oxygen soil environments by providing an internal aeration system for the transfer of oxygen from the shoot. However, aerenchyma formation requires Programmed Cell Death (PCD) in the root cortex (Drew et al., 2000; Bartoli et al., 2015; Fujimoto et al., 2018; Guan et al., 2019). Interestingly, both the aerenchyma formation and PCD in waterlogged sunflower stems are promoted by ethylene and ROS (Steffens et al., 2011; Petrov et al., 2015; Ni et al., 2019). In the root, during lysigenous aerenchyma formation under oxygen-deficient conditions, the precise balancing of ROS production and scavenging serves a crucial role (Paradiso et al., 2016; Yamauchi et al., 2017a; Yamauchi et al., 2017b).

## Conclusions

Root system growth depends on maintaining the balance between root-tip cell proliferation and differentiation (Petricka et al., 2012). In the meristematic zone, the cells exhibit higher rates of cell division, but they do not elongate; in the elongation zone, the cells cease to proliferate, become elongated, and start to differentiate (Beemster and Baskin, 1998). The maturation zone is characterized by fully elongated cells that undergo differentiation to form different types of cells, including root hairs (Caño-Delgado et al., 2010; Mendrinna and Persson, 2015). More importantly, the lateral roots are developed from primary roots in the maturation zone. These newly formed organs are important for the branching structure of the root system (Vermeer and Geldner, 2015). Research on the components that regulate such a balance is crucial to understanding plant growth and root development (Tsukagoshi, 2016). After decades of research, several pivotal plant hormones involved in root development have been identified (Ubeda-Tomas et al., 2009; Vanstraelen and Benková, 2012; Yamada and Sawa, 2013; Schaller et al., 2015). Recent studies have shown that ROS can function as signaling molecules to regulate root system growth (Mhamdi and Van Breusegem, 2018; Waszczak et al., 2018; Biswas et al., 2019; Chapman et al., 2019; Trevisan et al., 2019). ROS are especially important in maintaining the balance between cell proliferation and differentiation. The hypothesis that ROS have a hormone-like function by acting as signaling molecules is supported by a substantial amount of evidence (Yang et al., 2018; Mhamdi and Van Breusegem, 2018; Waszczak et al., 2018). From these results, ROS appear to be key to vital processes, including stem-cell maintenance, cell-cycle progression, and root hair initiation in the maturation zone of roots. Future research should aim to further elucidate the involvement of ROS in these processes. This will advance our understanding of the role of ROS in root development (Boisson-Dernier et al., 2013).

## Author Contributions

XMZ carefully revised and edited the manuscript and replot the figures. YX wrote the draft of the manuscript and CLL performed a part of the experiments in the manuscript, and GHY revised, guided, and improved the manuscript.

## Funding

This work was supported by the Special Fund for Basic Scientific Research of Central Colleges, South-Central University for Nationalities (CZP17051), National Natural Science Foundation of China (31270361), and Fund for Key Laboratory Construction of Hubei Province (Grant No.2018BFC360). It was also partially supported by the Regulation Mechanism Studies on Agronomic Traits in Important Crops and *Arabidopsis thaliana* aided by the State Administration of Foreign Experts Affairs in Ministry of Science and Technology of the People’s Republic of China (P193009007).

## Conflict of Interest

The authors declare that the research was conducted in the absence of any commercial or financial relationships that could be construed as a potential conflict of interest.
